# Graphene *versus* Multi-Walled Carbon Nanotubes for Electrochemical Glucose Biosensing

**DOI:** 10.3390/ma6031011

**Published:** 2013-03-14

**Authors:** Dan Zheng, Sandeep Kumar Vashist, Michal Marcin Dykas, Surajit Saha, Khalid Al-Rubeaan, Edmond Lam, John H.T. Luong, Fwu-Shan Sheu

**Affiliations:** 1 NUSNNI-NanoCore, National University of Singapore, T-Lab Level 11, 5A Engineering Drive 1, 117580, Singapore; E-Mails: zhengdan@nus.edu.sg (D.Z.); skvashist2007@gmail.com (S.K.V.); michal.dykas@nus.edu.sg (M.M.D.); physuraj@nus.edu.sg (S.S.); 2 Department of Chemistry, National University of Singapore, 3 Science Drive 3, 117543, Singapore; 3 Department of Electrical and Computer Engineering, National University of Singapore, Engineering Drive 1, 117576, Singapore; 4 NUS Graduate School of Integrative Sciences and Engineering, National University of Singapore, 28 Medical Drive, 117456, Singapore; 5 Department of Physics, National University of Singapore, 2 Science Drive 3, 117572, Singapore; 6 University Diabetes Center, King Saud University, P. O. Box 18397, Riyadh 11415, Saudi Arabia; E-Mail: krubean@ksu.edu.sa; 7 National Research Council Canada, Montreal, Quebec H4P 2R2, Canada; E-Mails: edmond.lam@cnrc-nrc.gc.ca (E.L.); john.luong@cnrc-nrc.gc.ca (J.H.T.L.)

**Keywords:** graphene, multi-walled carbon nanotubes, electrochemical glucose sensor, glucose oxidase

## Abstract

A simple procedure was developed for the fabrication of electrochemical glucose biosensors using glucose oxidase (GOx), with graphene or multi-walled carbon nanotubes (MWCNTs). Graphene and MWCNTs were dispersed in 0.25% 3-aminopropyltriethoxysilane (APTES) and drop cast on 1% KOH-pre-treated glassy carbon electrodes (GCEs). The EDC (1-ethyl-(3-dimethylaminopropyl) carbodiimide)-activated GOx was then bound covalently on the graphene- or MWCNT-modified GCE. Both the graphene- and MWCNT-based biosensors detected the entire pathophysiological range of blood glucose in humans, 1.4–27.9 mM. However, the direct electron transfer (DET) between GOx and the modified GCE’s surface was only observed for the MWCNT-based biosensor. The MWCNT-based glucose biosensor also provided over a four-fold higher current signal than its graphene counterpart. Several interfering substances, including drug metabolites, provoked negligible interference at pathological levels for both the MWCNT- and graphene-based biosensors. However, the former was more prone to interfering substances and drug metabolites at extremely pathological concentrations than its graphene counterpart.

## 1. Introduction

Graphene has been widely used for the development of optoelectronic devices [1], super capacitors [2] and various types of high performance sensors [3,4,5,6,7] due to its high surface-area-to-volume ratio [8,9], excellent electrical conductivity and high electron mobility [10]. Graphene, with a large surface area, enhances the loading of biomolecules by passive adsorption or covalent crosslinking, while its excellent conductivity and small band gap are beneficial for the conduction of electrons between the biomolecule and the electrode surface [10]. It has been claimed that graphene may not be beneficial as an electrode material, due to its lower edge surface area, leading to slow heterogeneous electron transfer [11]. The surface coverage and orientation of graphene on the electrode may also significantly affect its electrochemical performance [12].

It is of considerable interest to evaluate if graphene is advantageous compared to carbon nanotubes (CNTs) in various applications; particularly, in electrochemical biosensing for glucose, since the latter, with a high surface-volume ratio, has been extensively used in the development of super capacitors [13,14,15], energy storage devices [16], environmental sensing devices [17,18], drug delivery systems [19], biosensors [20,21] and other devices. The literature also offers several reviews discussing the comparison of graphene- and CNT-based electronic devices [15,22,23], hydrogen physical adsorption [24], chemical sensors/biosensors [25] and fuel cells [26]. The thermal properties [27], energy dispersion [28], electrical properties [29,30] and photocatalytic properties [31] of these materials have also been compared. However, there are only a few reports where graphene and CNT-based electrodes are compared for various biosensors [32,33,34], and to our knowledge, there is no comparison of graphene- and multi-walled carbon nanotube (MWCNT)-based electrodes for electrochemical glucose biosensing with respect to direct electron transfer.

**Scheme 1:**
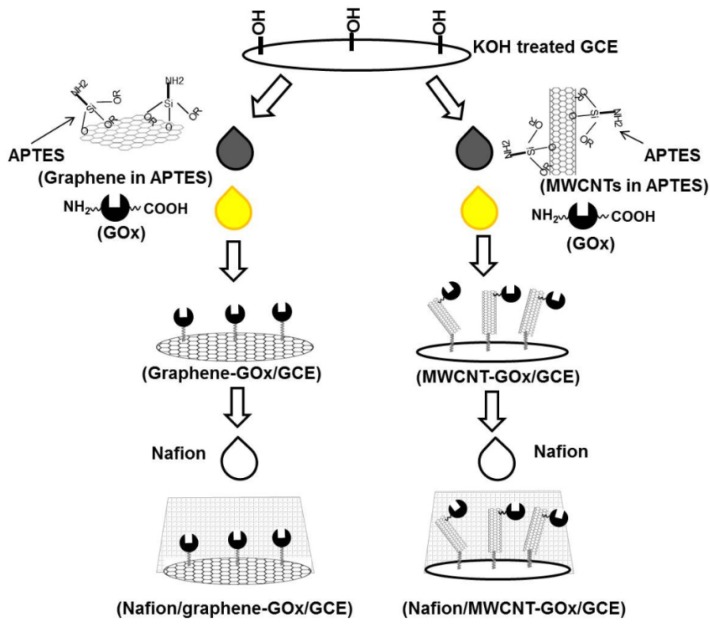
The preparation of graphene- and multi-walled carbon nanotube (MWCNT)-based glucose biosensors.

This study describes a simple procedure for the fabrication of the graphene- and MWCNT-based electrochemical glucose biosensors using glucose oxidase (GOx) (Scheme 1). The GOx covalently bound to graphene- or MWCNT-based electrodes will be evaluated for its direct electron transfer (DET) with the underlying electrode. The analytical performance of both biosensors will also be compared with respect to detection limit, linearity and interference caused by potential interfering substances and drug metabolites at normal and extreme physiological levels. 

## 2. Results and Discussion

### 2.1. Development of GOx-Bound Graphene- and MWCNT-Functionalized Glassy Carbon Electrodes (GCEs)

3-aminopropyltriethoxysilane (APTES) is used extensively as a surface modification agent for generating free amino groups on various bioanalytical platforms. The amino group of APTES is then bound to biomolecules, including antibodies, by 1-ethyl-(3-dimethylaminopropyl) carbodiimide (EDC)-sulfo-*N*-hydroxysuccinimide-based crosslinking [35,36,37]. In this study, APTES was used for dispersion, as well as surface modification for graphene [38] and MWCNTs [39]. GOx-bound graphene- or MWCNT-functionalized GCEs were prepared by a simple procedure (Scheme 1) and employed for mediatorless amperometric glucose biosensing. In brief, the procedure is based on the initial binding of the alkoxy groups of APTES to the hydroxyl groups on the KOH-treated GCE and graphene/MWCNTs. The treatment of a thin cell GCE (3 mm in diameter, BASi, MF-1000, West Lafayette, IN, USA) with KOH resulted in a slight increase in oxygen content associated with the formation of hydroxyl. SEM-EDX analyses revealed that the KOH-treated GCE exhibited 94.8% C and 5.2% O, compared to 95.5% C and 4.5% obtained for the untreated GCE. The dispersion of graphene/MWCNTs in APTES leads to their functionalization with APTES, while the unbound APTES molecules also functionalize the GCE’s surface. Thereafter, the formation of siloxane bonds (–Si–O–Si–) between the APTES molecules conjugated on graphene/MWCNTs and on the GCE leads to the preparation of graphene-/MWCNT-functionalized GCE. This is followed by the subsequent crosslinking of the free amino groups of APTES on the graphene-/MWCNT-functionalized GCE to the EDC-activated GOx. Finally, the graphene-GOx/GCE and MWCNT-GOx/GCE were covered with 0.5% Nafion to serve as a glucose limiting membrane. 

**Figure 1:**
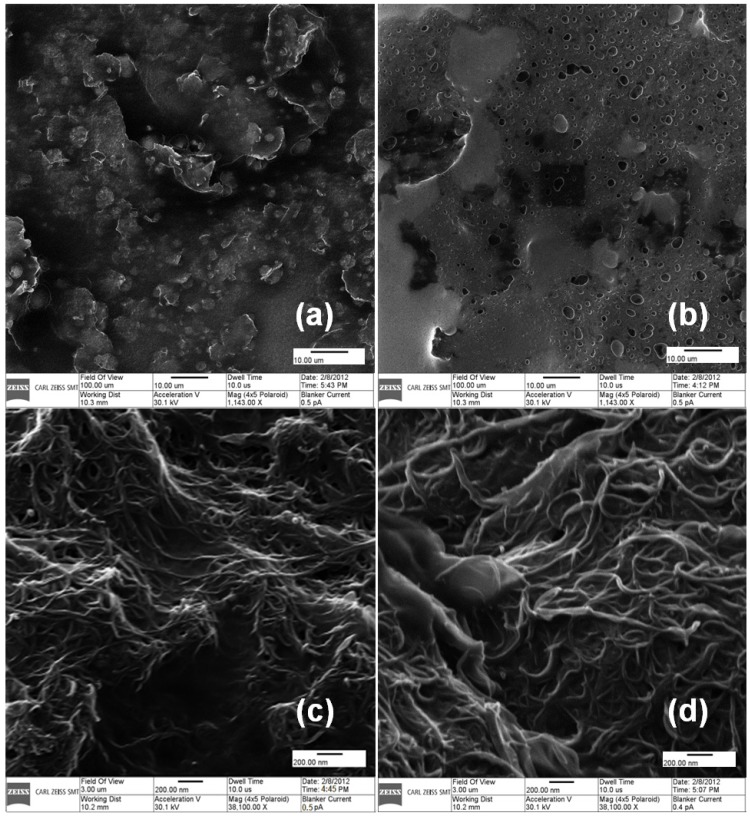
High resolution images of (**a**) grapheme-glucose oxidase (GOx); (**b**) Nafion/graphene-GOx; (**c**) MWCNT-GOx and (**d**) Nafion/MWCNT-GOx modified glassy carbon substrates using a helium ion microscope from Carl Zeiss, Germany. The scale bars for (**a**)/(**b**) and (**c**)/(**d**) are 10 μm and 200 nm, respectively.

The carbon material-enzyme mixtures (Figure 1a,b) were spread uniformly on glassy carbon. The coating of carbon material-GOx mixtures with Nafion changed their appearance (Figure 1b,d), which confirmed the covering of the carbon material-GOx functionalized GCEs with a Nafion thin layer. As described in Figure S1 (Supplementary), Raman signatures of pristine MWCNTs (and graphene) exhibit peaks near 1350, 1580, 1620, 2450, 2700, 3168 and 3238 cm^−1^, known as D, G, D′, G*, 2D, D + G and 2D′ peaks, respectively. These are characteristics of defects and atomic vibrations in the carbon network [40,41]. However, the Raman spectrum of APTES functionalized MWCNTs (and graphene) shows a red-shift (*i.e.*, peaks shift to lower energy) of all the characteristic peaks, suggesting an n-type doping of MWCNTs (and graphene), revealing the functionalization of the carbon materials by APTES [42]. The red-shift observed in graphene was comparatively lesser than in MWCNTs. This may not be due to electron/hole doping, but a possible tiny-change in the effective mass of the graphene unit cell in APTES functionalized graphene. Nevertheless, the red-shift in the Raman signatures of graphene itself is suggestive of functionalization by APTES. 

The reaction intermediate of each step in Scheme 1 was also characterized by FTIR to confirm the immobilization of GOx on such modified electrodes. The KOH-treated GCE (3 mm in diameter, BASi, MF-1000, West Lafayette, IN, USA) functionalized with APTES exhibited several important FTIR bands, including 1556 and 1484 cm^−1^ (ν(NH_2_)), 1433 cm^−1^ (ν_s_(CH_3_)), 1383 cm^−1^ (ν_as_(CH_3_)) and 1371 cm^−1^ (ν(CH_2_ backbone)). Further modification of this electrode with MWCNTs (dispersed in dimethylformamide) shows new FTIR bands at 1141 cm^−1^ (ν_as_(C-O)), 2976 cm^−1^ (ν_s_(CH_2_)) and 3015 cm^−1^ (ν_as_(CH_2_)), which could be attributed to defect sites on the CNT surface (Figure S2, curve a, Supplementary). This FTIR signature was identical to the one obtained for the GCE modified with MWCNTs dispersed in APTES (Figure S2, curve b). Figure S2 (curve c) shows the characteristic amide I and amide II bands of GOx centered at 1655 cm^−1^ and 1535 cm^−1^, the most compelling evidence for the immobilization of GOx on the MWCNT/APTES-functionalized GCE. 

The FTIR spectra for graphene deposited on GCE (pretreated with KOH) using a solution of graphene dispersed in APTES or layer by layer APTES then graphene in dimethylformamide (DMF) were very similar (Figure S3, curves a and b). No significant peak was observed for graphene, except for two small peaks at 1565 (skeletal vibration of graphene sheet) and 1150 cm^−1^. Similar features for Si–O–C and Si–O–Si bonds were found in the 1000–1100 cm^−1^ range. When GOx activated with EDC was added to the composite, the presence of bands at 1638 and 1521 cm^−1^ confirmed that GOx was bound to the APTES through amide linkages (Figure S3, curve c).

### 2.2. Evaluation of Direct Electron Transfer 

Cyclic voltammetry (CV) was performed on the graphene- and MWCNT-functionalized GCEs at varying scan rates (20–200 mV s^−1^) in 5 mM potassium ferricyanide (K_3_Fe(CN)_6_, in 0.5 M KCl) (Figure S4, Supplementary). The peak potential (*E*_p_) remained almost unchanged at varied scan rates for both electrodes. The cathodic/anodic peak current (*i*_pc_ and *i*_pa_) plotted against the square root of the scan rate was linear, confirming the reversible redox reaction of the Fe(CN)_6_^3−^/Fe(CN)_6_^4−^ couple on graphene- and MWCNT-functionalized GCEs. For a reversible process [43]:

*i*_p_ = (2.69 × 10^5^)*n*^3/2^*AD*_O_^1/2^*v*^1/2^*C*_O_^*^ (1)

where *n* is the number of electrons transferred, *A* is the effective area, *D*_O_ is the diffusion coefficient of Fe(CN)_6_^3−^, *v* is the scan rate and *C*_O_ is the bulk concentration of Fe(CN)_6_^3−^. With 5 mM Fe(CN)_6_^3−^ in 0.5 M KCl, *n* = 1, *D*_O_ = 7.6 × 10^−6^ cm^2^ s^−1^, the effective surface area of the graphene/GCE and the MWCNT/GCE was estimated to be 0.072 and 0.11 cm^2^, respectively.

**Figure 2:**
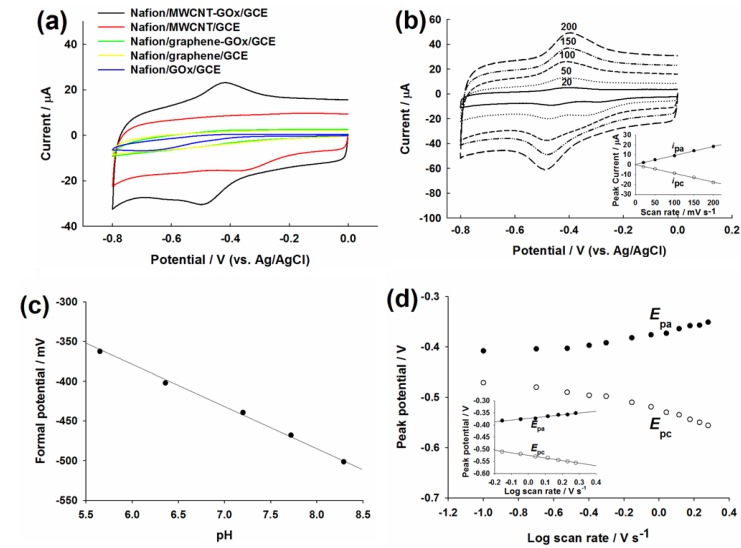
(**a**) CVs of Nafion/GOx/glassy carbon electrodes (GCE) (blue), Nafion/graphene/GCE (yellow), Nafion/MWCNT/GCE (red), Nafion/graphene-GOx/GCE (green) and Nafion/MWCNT-GOx/GCE (black) in N_2_-saturated PBS at 100 mV s^−1^; (**b**) The effect of scan rate (20, 50, 100, 150 and 200 mV s^−1^) on the DET of GOx on Nafion/MWCNT-GOx/GCE in N_2_-saturated PBS. Inlet: the linear relation between *i*_pc_ (or *i*_pa_) and *v*; (**c**) The relation between the formal potential (observed on Nafion/MWCNT-GOx/GCE) and different pH values: 5.65, 6.36, 7.2, 7.72, 8.29. Scan rate = 100 mV s^−^^1^; (**d**) Plot of *E*_p_ (of the Nafion/MWCNT-GO*x*/GCE) *vs.* log *v*, *v* = 0.1, 0.2, 0.3, 0.4, 0.5, 0.7, 0.9, 1.1, 1.3, 1.5, 1.7, 1.9 V s^−^^1^. Inlet: the relation between *E*_pa_ (or *E*_pc_) and log *v*.

Figure 2a shows the CVs of Nafion/GOx/GCE (blue), Nafion/graphene/GCE (yellow), Nafion/MWCNT/GCE (red), Nafion/graphene-GOx/GCE (green) and Nafion/MWCNT-GOx/GCE (black) in nitrogen (N_2_)-saturated 50 mM PBS at 100 mV s^−1^. Rectangle-shaped CVs were observed on the Nafion/graphene/GCE (yellow) and the Nafion/MWCNT/GCE (red) in the applied potential range. The double-layer capacitance of Nafion/MWCNT/GCE was significantly greater than Nafion/graphene/GCE. 

The redox peaks of FAD/FADH_2_ were not observed for Nafion/graphene-GOx/GCE (green). In contrast, a pair of well-defined redox peaks were observed on the Nafion/MWCNT-GOx/GCE (black) with the cathodic peak potential (*E*_pc_) of −0.49 V and an anodic peak potential (*E*_pa_) of −0.42 V. Therefore, the formal potential (*E*^0^) was −0.455 V, which reflects the typical electrochemical characteristics of GOx immobilized on CNT-based electrodes in neutral solution [44,45]. The cathodic peak is attributed to the reduction of FAD to FADH_2_, while the anodic peak is due to the reversible re-oxidization of FADH_2_ to FAD [46]. The DET of GOx observed on the MWCNT-modified electrode may be facilitated by the three dimensional structure of MWCNTs that results in a shortened tunneling distance for the electron transfer between the enzyme and the underlying electrode surface [46]. As the electrochemical property or the structure of commercial graphene is very different from that of MWCNTs, the DET between GOx and the electrode surface cannot be observed by the simple CV approach.

The scan rate effect on the electrochemical response on Nafion/MWCNT-GOx/GCE is shown in Figure 2b. Notice that two separate reduction peaks were observed on the electrode at about −0.3 V and −0.47 V at 20 and 50 mV s^−1^. The reduction peak at about −0.47 V should be due to the reduction of GOx-FAD to form GOx-FADH_2_, but the unexpected one at −0.3 V may be owing to the metallic impurity in MWCNTs. Indeed, the reduction peak at −0.3 V was also observed on the Nafion/MWCNT/GCE (Figure 2a, red). However, only the reduction peak of GOx-FAD was observable at scan rates higher than 50 mV s^−^^1^ (100, 150 and 200 mV s^−^^1^). The linear increase of *i*_pc_ and *i*_pa_ with increasing scan rate from 20 to 200 mV s^−1^ confirmed the excellent electrocatalysis of MWCNTs and the redox reaction of FAD/FADH_2_ couple on MWCNT as a surface-controlled electrochemical process [47,48]. 

The pH effect on the electrochemical behavior of GOx at the Nafion/MWCNT-GOx/GCE was also studied, as shown in Figure 2c. The formal potential of the Nafion/MWCNT-GOx/GCE varied linearly with pH, varying from 5.65 to 8.29. The slope of the formal potential *vs.* pH was −51.6 mV/pH, close to the theoretical value of −59 mV/pH for a two-electron coupled with two-proton redox reaction [44], as shown in Equation (2). The charge transfer coefficient, *α*, and the heterogeneous transfer rate constant, *k*_s_, were then estimated based on the Laviron’s model (for Δ*E*_p_ > 0.200 V, Equation (3)) [49,50]:

GOx-FAD + 2e^−^ + 2H^+^ ↔ GOx-FADH_2_ (2)

(3) $$\text{log}k_{s}=\alpha \log\left(1-\alpha \right)+\left(1-\alpha \right)\text{log}\alpha -\text{log}\frac{RT}{nFv}-\frac{\alpha (1-\alpha )nF\Delta E_{p}}{2.3RT}$$

where *n* is the number of electrons transferred in the rate-determining reaction, Δ*E*_p_ is the peak-to-peak potential difference and *v* is the scan rate. The plot of *E*_p_ *vs.* log *v* (Figure 2d) exhibited two straight lines with a slope of −2.3*RT*/*αnF* and 2.3*RT*/(1 − *α*)*nF* for the cathodic and anodic peaks, respectively. The average value of *α* and *k*_s_ was calculated to be 0.59 and 2.05 s^−1^, respectively. The *k*_s_ is higher than the results reported for GOx immobilized in CNTs (1.78 s^−1^ [51] and 1.69 s^−1^ [52]) or gold nanoparticle incorporated matrices (1.69 s^−1^ [53]). 

### 2.3. Evaluation of Glucose Oxidation

**Figure 3:**
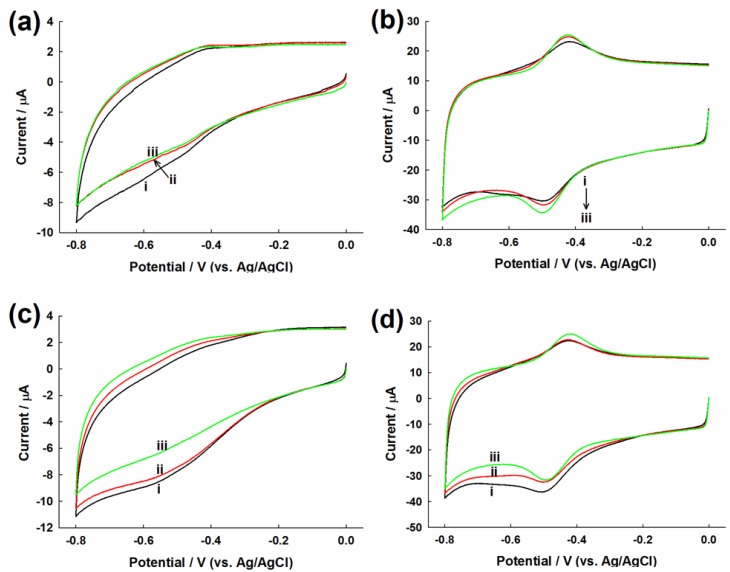
CVs of (**a**,**c**) Nafion/graphene-GOx/GCE and (**b**,**d**) Nafion/MWCNT-GOx/GCE in (**a**,**b**) nitrogen and (**c**,**d**) air-saturated PBS containing (i) 0 mM; (ii) 1 mM and (iii) 8 mM glucose. Scan rate: 100 mV s^−1^.

In N_2_-saturated glucose solution, the cathodic currents of Nafion/graphene-GOx/GCE decreased with increasing glucose concentration from 0 to 8 mM (Figure 3a). In contrast, both the *i*_pc_ and *i*_pa_ of Nafion/MWCNT-GOx/GCE increased when glucose was increased from 0 to 8 mM (Figure 3b). The increase of *i*_pc_ suggested that the direct electrochemical reduction of FAD to FADH_2_ was enhanced with the increase in glucose concentrations, resulting in the build-up of FADH_2_ that led to increased *i*_pa_. According to a previous report, only increasing *i*_pa_ of GOx is observed on the GCE decorated with a hollow structured polymer-nickel oxide composite [46]. 

The cathodic currents of Nafion/graphene-GOx/GCE for various glucose concentrations under air-saturated condition (Figure 3c) exhibited a similar tendency to those under the N_2_-saturated condition. The elucidation of an exact mechanism for the electrochemistry of GOx on Nafion/graphene-GOx/GCE in the absence and presence of oxygen requires further research efforts. In contrast, the cathodic peak currents decreased with the increase in glucose concentration on Nafion/MWCNT-GOx/GCE (Figure 3d), which may be due to the GOx-catalyzed oxygen reduction on the GCE [46,47]. The reduction wave corresponds to the FADH_2_-GOx catalyzed reduction of O_2_. As O_2_ is consumed by FADH_2_, its concentration at the electrode surface is reduced, resulting in the decrease in reduction current with the increase in glucose concentration. Nevertheless, the *i*_pa_ did not change appreciably for various glucose concentrations. Without the enzyme, the control electrodes, *i.e.*, Nafion/graphene/GCE and Nafion/MWCNT/GCE, did not show any change in the current signal with varying glucose concentration, both in the absence and presence of O_2_ (data not shown).

### 2.4. Amperometric Detection of Commercial and Blood Glucose

**Figure 4:**
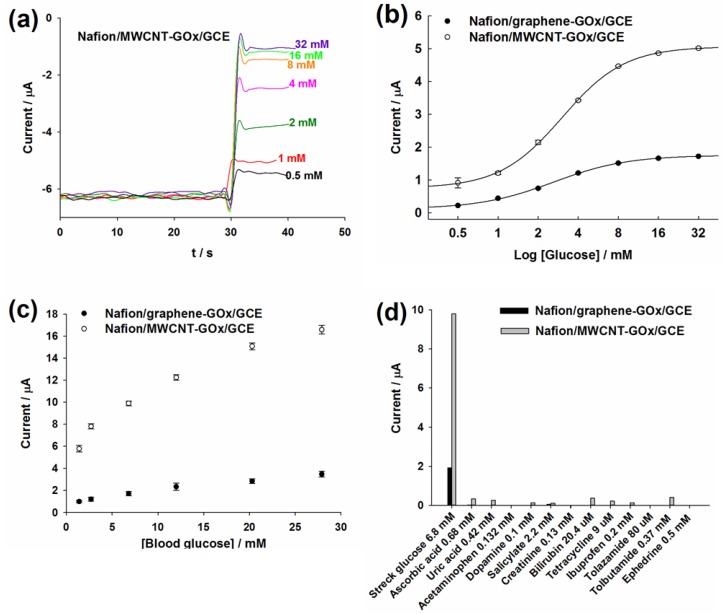
(**a**) The amperometric response of Nafion/MWCNT-GOx/GCE for the detection of 0.5 to 32 mM glucose at −0.45 V in the presence of O_2_; (**b**) Assay curves for the detection of commercial glucose by the graphene- and MWCNT-based electrodes. The error bars represent standard deviation (SD); (**c**) Assay curves for the detection of Sugar-Chex whole blood glucose linearity standards by both electrodes. The error bars represent the SD; (**d**) The effect of interfering substances on the electrochemical detection of 6.8 mM blood glucose standard by both electrodes.

As both *in vivo* and *in vitro* blood samples contain dissolved oxygen, the decrease of the cathodic current on Nafion/graphene-GOx/GCE and Nafion/MWCNT-GOx/GCE can be used to detect glucose by the amperometric *i*–*t* curve in the presence of O_2_. The Nafion coating was employed to circumvent limited oxygen concentration in PBS, as it acts as a glucose limiting membrane to prevent excess glucose molecules from being converted by GOx. The optimum applied potential was −0.45 V (Supplementary, Figure S5). Figure 4a illustrates the amperometric response of the MWCNT-based electrode for detecting 0.5~32 mM commercial glucose. Figure 4b shows the assay curves for glucose detection by GOx-bound graphene- and MWCNT-functionalized GCEs. Both electrodes exhibited dynamic responses to varying glucose concentrations up to 16 mM. However, the current response of commercial glucose detected by Nafion/MWCNT-GOx/GCE was >2-fold higher than that of Nafion/graphene-GOx/GCE. Considering the higher effective surface area of MWCNT/GCE (0.11 cm^2^) *versus* 0.072 cm^2^ for graphene/GCE, GOx apparently exhibited higher activity on the Nafion/MWCNT substrate. Note that the glucose linear range for both graphene- and MWCNT-based glucose biosensors was 0.5–4 mM. The detailed comparison between this work and recently reported graphene- and CNT-based glucose biosensors is shown in Table 1. 

**Table 1:** Detailed comparison between this work and recently reported graphene- and CNT-based glucose biosensors.

Graphene- or CNT-based Glucose Biosensor	Glucose Linear Range (mM)	Real Sample Study	Interfering Study	Reference
Nafion/graphene-GOx/GCE	0.5–4 (dynamic range: 0.5–16)	Detect 1.4–27.9 mM blood glucose in diluted Streck samples	No interference from physiological levels of interfering substances	This work
Graphene oxide-chitosan-GOx/GCE	4 × 10^−4^–2	Detect 5 and 10 mM glucose added into serum samples	No interference from 2 mM ascorbic acid, uric acid, citric acid and acetaminophen; not testing for other interfering substances	[54]
Carboxyl-long-chain-graphene oxide modified with Fe_3_O_4_, polyaniline and GOx	1–1.4	Detect blood glucose (0.2–1.4 mM) in diluted serum samples	No interference from 0.3 mM ascorbic acid and uric acid and 0.01 mM immunoglobulin G; not testing for other interfering substances	[55]
Palladium nanoparticle/chitosan-grafted graphene/GCE	1 × 10^−3^–1	Detect blood glucose in diluted blood samples (recovery: 92.5%–105.3%)	No interference from 0.2mM ascorbic acid and 0.5 mM uric acid; not testing for other interfering substances	[56]
Nafion/MWCNT-GOx/GCE	0.5–4 (dynamic range: 0.5–16)	Detect 1.4–27.9 mM blood glucose in diluted Streck samples	Negligible interference from interfering substances	This work
A mixture of GOx and a CNT film sandwiched with 10 nm thick PPFs	0.025–2.2	Not testing for real samples	No interference from 0.5 mM ascorbic acid	[57]
Incorporation of GOx into the colloidal Au-CNT composite matrix	0.05–1	Not testing for real samples	No interference from 1 μM cysteine and 0. 1 μM uric acid; significant interference from 1 μM ascorbic acid; not testing for other interfering substances	[58]
GOx-platinum nanoparticle-CNT-titania nanotube array modified electrode	6 × 10^−3^–1.5	Not testing for real samples	Not testing for the effect of interfering substances	[59]

The Sugar-Chex whole blood glucose linearity standards from Streck (US) were employed to evaluate the sensing performance of the developed biosensors for the detection of blood glucose. Both the developed electrodes were able to detect 1.4–27.9 mM glucose that covers the entire pathophysiological range of glucose in diabetics (Figure 4c). However, the current response for the Nafion/MWCNT-GOx/GCE was >4-fold higher than that of Nafion/graphene-GOx/GCE, thereby illustrating the superior analytical performance of MWCNTs for the development of electrochemical glucose biosensor. The higher current signal provided by Nafion/MWCNT-GOx/GCE could be attributed to the larger effective surface area of MWCNT/GCE that leads to higher GOx immobilization.

### 2.5. Effect of Interfering Substances

The interference was determined as the percentage of the current signal, obtained for detecting a specific concentration of blood glucose, which was contributed by the addition of a particular interfering substance. The interfering substances with pathophysiological concentrations, about 2–20-fold higher than their physiological concentrations, did not induce any considerable interference to the electrochemical detection of 6.8 mM blood glucose by Nafion/graphene-GOx/GCE. Bilirubin (0.34 mM) or 3.7 mM tolbutamide only induced less than 5.2% interference, while 3.62 mM salicylate, as well as 3.21 mM tolazamide induced about 4% interference. Except for 0.5 mM ephedrine, the remaining interfering species only resulted in <3% error in the detection of blood glucose. However, there was no interference from these interfering substances at their physiological concentrations (Figure 4d). For Nafion/MWCNT-GOx/GCE, the pathophysiological concentrations of interfering substances and drugs caused appreciable interferences, resulting in higher current signals (data not shown). However, the interference was significantly reduced when such interfering substances were tested at their corresponding physiological levels (Figure 4d). Bilirubin (20.4 μM) and 0.68 mM ascorbic acid only induced about 3.6% interference, compared with 4% for 0.37 mM tolbutamide. There was no interference from 0.13 mM creatinine, while 0.132 mM acetaminophen and 0.5 mM ephedrine, the remaining interfering substances, had about less than 2.5% interference. Work is in progress to investigate long-term storage stability, anti-biofouling, production reproducibility and robustness of functional GOx immobilization of the graphene- and MWCNT-based glucose biosensors.

## 3. Experimental Section 

### 3.1. Chemicals

Graphene was purchased from Cheap Tubes (USA, diameter 5 μm) and used as received. MWCNTs (diameter 15 ± 5 nm and length 1–5 µm, purity >95%) were bought from NanoLab (MA, USA). GOx (EC 1.1.3.4, Type X-S from *Aspergillus niger*, G7141), D-glucose, 5 wt % Nafion, K_3_Fe(CN)_6_, KCl, APTES, glutaraldehyde, dimethylformamide (DMF) and all interfering substances (electrochemical active drugs) were purchased from Sigma-Aldrich. BupH phosphate buffered saline (PBS), BupH MES buffered saline and EDC were procured from Fisher Thermo Scientific. Sugar-Chex Linearity (whole blood glucose linearity standards) was purchased from Streck, Inc. (USA). The dilutions of APTES and glutaraldehyde were made in ultrapure water (18.2 MΩ cm at 25 °C, Direct Q, Millipore), whereas GOx and glucose were dissolved and prepared in 50 mM PBS. EDC was prepared in 100 mM MES (pH 4.7), and the dilution of Nafion to 0.5% was made in absolute ethanol. The GOx stock solution, prepared by mixing equal volumes of 20 mg mL^−1^ GOx and 5% glutaraldehyde, was stored overnight at 4 °C before use. The glucose solution was stored overnight at room temperature (RT), while the interfering substances were freshly prepared just before use.

### 3.2. Apparatus and Measurement

High resolution images of Graphene-GOx, MWCNTs-GOx, Nafion/graphene-GOx and Nafion/MWCNTs-GOx were taken by a helium ion microscope from Carl Zeiss, Germany. The Raman spectra of pristine graphene and MWCNTs, APTES-functionalized graphene and APTES-functionalized MWCNTs were recorded using a Renishaw micro-Raman system coupled to an air-cooled photomultiplier tube equipped with a 514.5 nm line of an Ar^+^-ion laser. Scanning electron microscopy (SEM)-energy dispersive X-ray (SEM-EDX) analysis was performed on a Hitachi S 2600N SEM (Hitachi Scientific Instruments, Tokyo, Japan) equipped with a microanalysis detector for EDX (Inca x-act, Oxford Analytical Instruments, Abington, UK). EDX spectra were collected at 30° angle, 20 kV accelerating voltage and 20 mm working distance. EDX results were analyzed using incorporated Inca, Point and Analyze software. Attenuated total reflectance FTIR (ATR-FTIR) spectra were collected from 4000 to 600 cm^−1^ for 64 scans and 4 cm^−1^ resolution using a zinc selenide (ZnSe) crystal on a Bruker Tensor 27 FTIR spectrophotometer. 

All electrochemical measurements were carried out at room temperature (RT) on the CHI 660A electrochemical workstation (CH Instruments, Austin, TX, USA) with a three-electrode system: graphene or MWCNT-based GO*x*-bound GCE as the working electrode, Pt wire counter electrode and Ag/AgCl (3M KCl) reference electrode. CV and amperometric *i*–*t* curve techniques were used for the electrochemical characterization of the biosensors. CV was also used to determine the effective surface area of MWCNT- and graphene-functionalized GCE in 5 mM K_3_Fe(CN)_6_ (dissolved in 0.5 M KCl). Unless otherwise specified, the electrochemical measurements were performed in the presence of oxygen dissolved in a reaction mixture under ambient air temperature. For CVs carried out in the nitrogen-saturated buffer, the electrolyte was bubbled with pure nitrogen for 30 min just before the experiments, and the nitrogen environment was maintained during electrochemical detection. All potentials were referred to Ag/AgCl.

### 3.3. Biosensor Fabrication

The fabrication of graphene- and MWCNT-based glucose biosensors followed a rapid and reagentless method developed by our group [60]. Typically, GCEs (3 mm diameter, CH Instruments, Austin, TX, USA) were polished consecutively by using 0.3 and 0.05 µm alumina powder and subsequently cleaned by putting in an ultrasonic bath for 20 min. The polished GCEs were then dipped in 1% KOH for 5 min to generate hydroxyl groups on their surface. Two microliters of 2 mg mL^−1^ graphene or MWCNTs (dispersed in 0.25% APTES) were drop-cast on the GCE, followed by immediate drop-casting of 2 μL of EDC activated-GOx (10 mg mL^−1^ GOx was mixed with 0.12 g mL^−1^ EDC in the volumetric ratio of 30:2 for 15 min at RT just before use). The graphene-GOx/GCE and MWCNT-GOx/GCE was dried at RT for 1 h and washed extensively with 50 mM PBS. Thereafter, they were drop-cast with 3 μL of 0.5% Nafion, dried at RT for 10 min and washed extensively with 50 mM PBS to form Nafion/graphene-GOx/GCE and Nafion/MWCNT-GOx/GCE. Nafion/graphene/GCE and Nafion/MWCNT/GCE were also prepared and employed as control.

### 3.4. Amperometric Glucose Detection

The detection of varying concentrations of glucose was done by injecting different volumes of 1 M glucose stock solution into stirred PBS to form 2 mL of 0.5, 1, 2, 4, 8, 16 and 32 mM glucose. All measurements of glucose were performed at each concentration level in triplicate samples. The assay curve of Sugar-Chex blood glucose linearity standards was obtained by injecting 400 μL of Sugar-Chex blood glucose linearity standards, having different glucose concentrations of 1.4, 2.7, 6.8, 12.0, 20.3 and 27.9 mM, into 2.8 mL of stirred PBS. The results obtained were then multiplied by the dilution factor.

### 3.5. Evaluating the Effect of Interferences on Glucose Detection

Bilirubin and uric acid solutions were prepared in 10 mM NaOH; creatinine, acetaminophen, ascorbic acid, dopamine and ephedrine solutions were prepared in 0.1 M PBS; ibuprofen, salicylate and tolbutamide solutions were prepared in absolute ethanol; the tetracycline solution was prepared in 3 M HCl; and the tolazamide solution was prepared in acetone. Thereafter, the effect of interfering substances on the specific electrochemical detection of 6.8 mM Sugar-Chex blood glucose linearity standard was determined.

## 4. Conclusions

The graphene- and MWCNT-based glucose biosensors were developed using a simple and rapid bioanalytical procedure. The DET of GOx was only observed by CVs in the case of MWCNTs, due to their electrocatalytic property. Nafion/MWCNT-GOx/GCE also provided >2- and 4-fold higher signals for commercial and blood glucose, respectively, in comparison to Nafion/graphene-GOx/GCE. The higher signal enhancement by MWCNTs may be due to their larger surface area, which leads to higher GOx loading. The exact molecular mechanism responsible for this behavior needs to be elucidated by further intensive research endeavors. Interfering substances and drug metabolites at their physiological concentrations exhibited no significant interference with the blood glucose determination in both biosensors. Apparently, MWCNTs served as a better electrode material compared to graphene for electrochemical glucose sensing using our developed biosensors. Notice also that some impurities in MWCNTs, particularly iron particles, can catalyze the oxidation of glucose. However, the electrochemical reaction of glucose only occurs at extreme alkali pH, e.g., in 0.1 M NaOH at >0.5 V applied potential [61]. Notice also that nanographite impurities in CNTs are also responsible for the electrochemical oxidation of tyrosine, tryptophan and NADH [62]. Again, the applied potential must be over +0.5 V *vs.* Ag/AgCl. Therefore, such impurities are not expected to cause any significant interference in the direct electron transfer between GOx, and the underlying electrode performed at neutral pH at −0.45 V applied potential.

## Supplementary Materials

Supplementary File 1
