# Supplementary material for: Graphene versus Multi-Walled Carbon Nanotubes for Electrochemical Glucose Biosensing
Source: Materials (Basel). 2013 Mar 14;6(3):1011–27. doi: 10.3390/ma6031011 (PMC5512961; doi:10.3390/ma6031011)
Supplement: Supplementary File 1 [file materials-06-01011-s001.pdf]

Supporting Information

Figure S1. Raman spectra of bare (red) and APTES-functionalized (blue) (**a**) graphene and (**b**) MWCNTs.

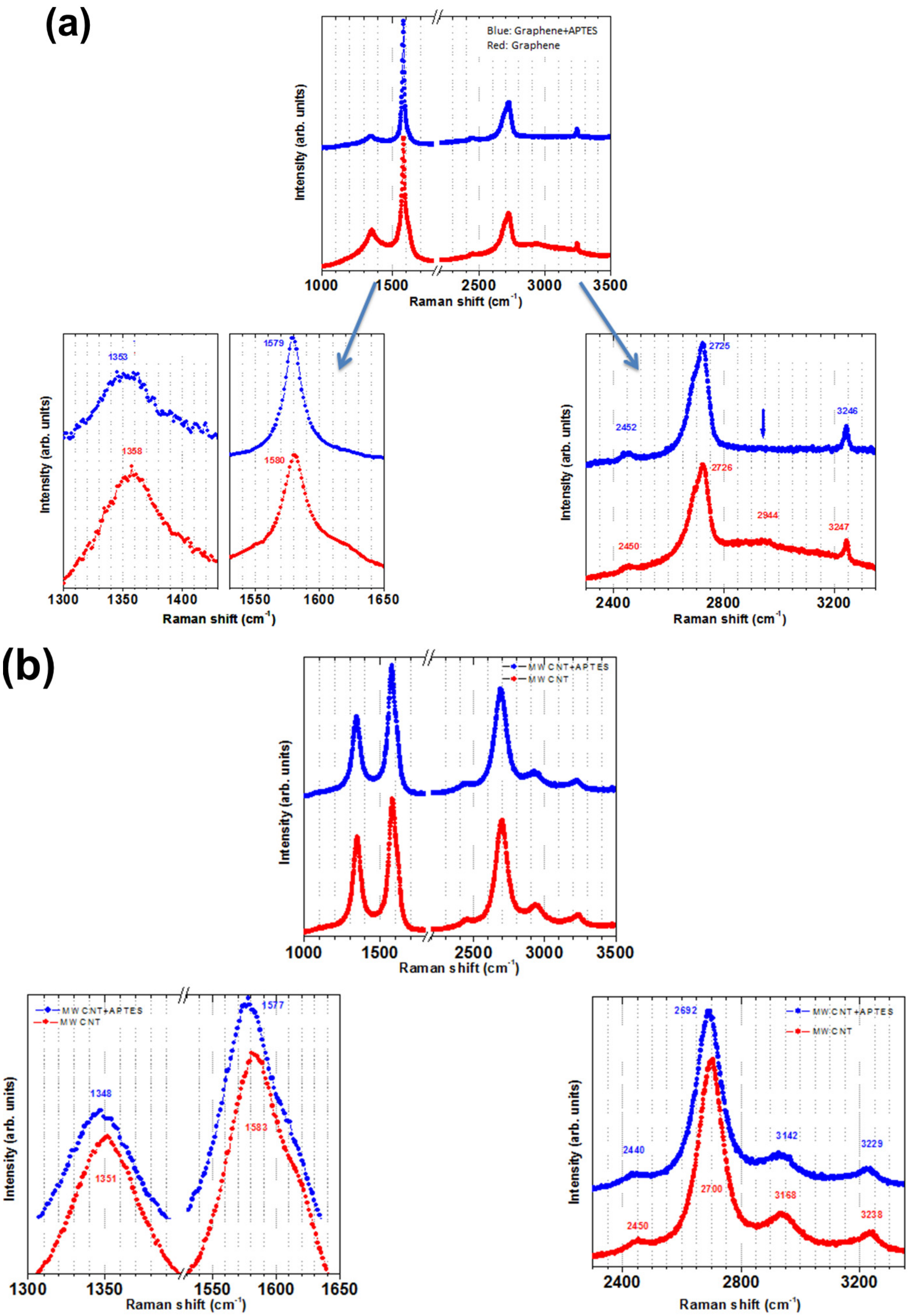

Figure S2. The FTIR spectrum of (**a**) the KOH-treated GCE modified with APTES and MWCNTs dispersed in dimethylformamide (DMF); (**b**) the KOH-treated GCE modified with MWCNTs dispersed in APTES; (**c**) same as (**b**) followed by conjugation with GO*x*.

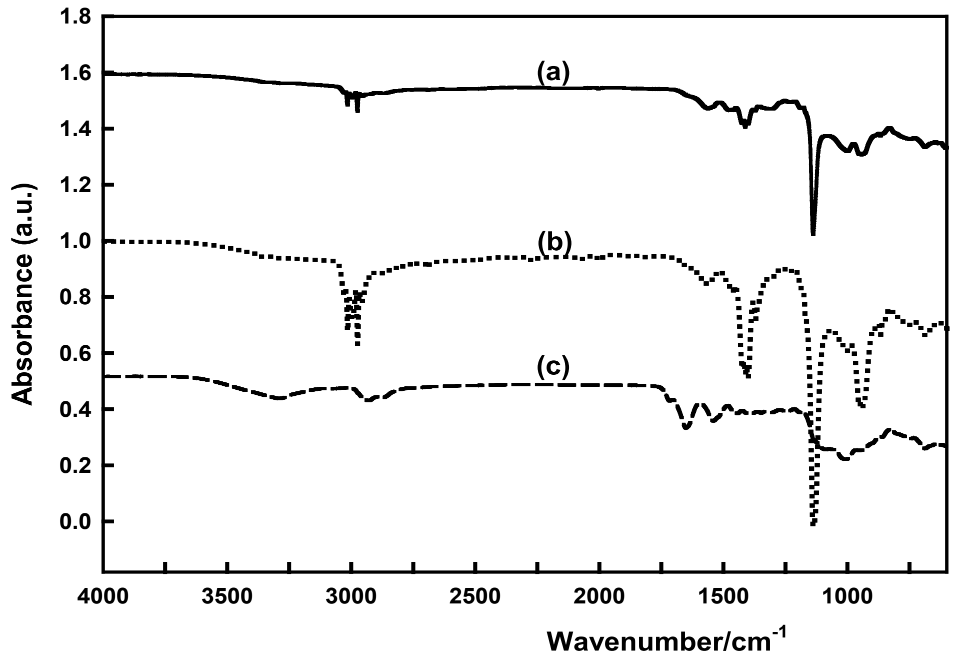

Figure S3. The FTIR spectrum of (**a**) the KOH-treated GCE modified with "layered by layered" APTES and graphene dispersed in DMF; (**b**) the KOH-treated GCE modified with graphene dispersed in APTES; and (**c**) same as (**b**) followed by conjugation with GO*x*.

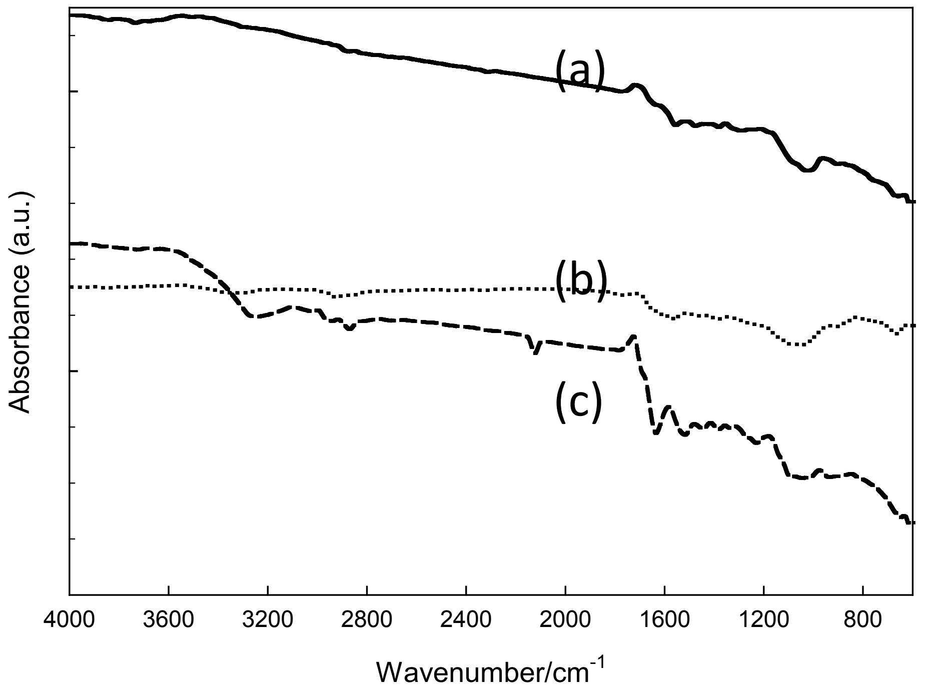

Figure S4. CVs of graphene- (**a**) and MWCNT-functionalized (**b**) GCE at different scan rates in 5 mM $Fe(CN)_6^{3-}$ (0.5 M KCl). Scan rate: 20–200 mV s^{-1}. Inlet: linear relationship between i_p and $v^{1/2}$.

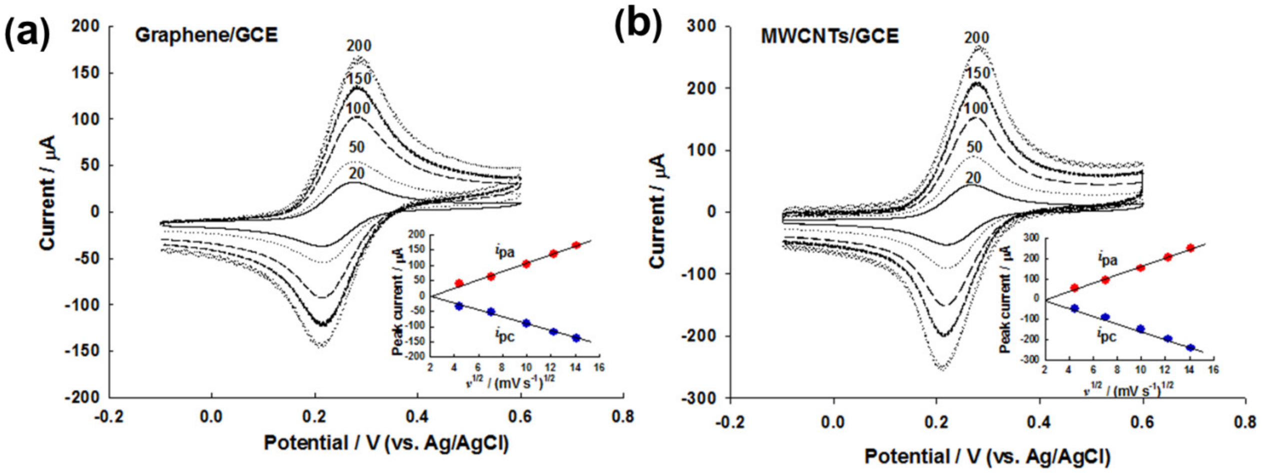

Figure S5. Optimization of the applied potential for the electrochemical detection of 4 mM glucose by Nafion/MWCNT-GO*x*/GCE.

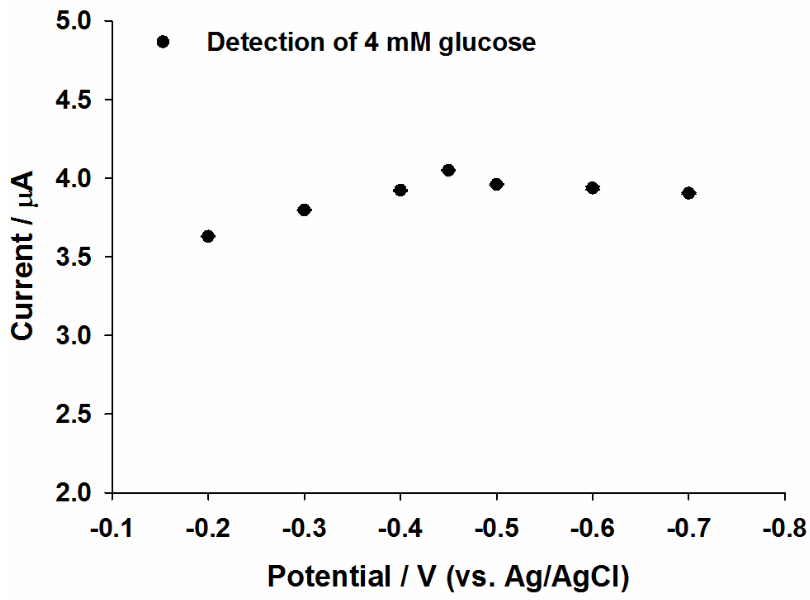

© 2013 by the authors; licensee MDPI, Basel, Switzerland. This article is an open access article distributed under the terms and conditions of the Creative Commons Attribution license (http://creativecommons.org/licenses/by/3.0/).
